# Flavonols as a Potential Pharmacological Intervention for Alleviating Cognitive Decline in Diabetes: Evidence from Preclinical Studies

**DOI:** 10.3390/life13122291

**Published:** 2023-11-30

**Authors:** Anđela Horvat, Ignacija Vlašić, Jasminka Štefulj, Nada Oršolić, Maja Jazvinšćak Jembrek

**Affiliations:** 1 Division of Molecular Medicine, Ruđer Bošković Institute, Bijenička 54, 10000 Zagreb, Croatia; 2 Division of Molecular Biology, Ruđer Bošković Institute, Bijenička 54, 10000 Zagreb, Croatia; 3 Department of Psychology, Catholic University of Croatia, Ilica 242, 10000 Zagreb, Croatia; 4 Division of Animal Physiology, Faculty of Science, University of Zagreb, Rooseveltov trg 6, 10000 Zagreb, Croatia

**Keywords:** diabetes, cognitive functions, flavonols, oxidative stress, neuroinflammation, signaling pathways

## Abstract

Diabetes mellitus is a complex metabolic disease associated with reduced synaptic plasticity, atrophy of the hippocampus, and cognitive decline. Cognitive impairment results from several pathological mechanisms, including increased levels of advanced glycation end products (AGEs) and their receptors, prolonged oxidative stress and impaired activity of endogenous mechanisms of antioxidant defense, neuroinflammation driven by the nuclear factor kappa-light-chain enhancer of activated B cells (NF-κB), decreased expression of brain-derived neurotrophic factor (BDNF), and disturbance of signaling pathways involved in neuronal survival and cognitive functioning. There is increasing evidence that dietary interventions can reduce the risk of various diabetic complications. In this context, flavonols, a highly abundant class of flavonoids in the human diet, are appreciated as a potential pharmacological intervention against cognitive decline in diabetes. In preclinical studies, flavonols have shown neuroprotective, antioxidative, anti-inflammatory, and memory-enhancing properties based on their ability to regulate glucose levels, attenuate oxidative stress and inflammation, promote the expression of neurotrophic factors, and regulate signaling pathways. The present review gives an overview of the molecular mechanisms involved in diabetes-induced cognitive dysfunctions and the results of preclinical studies showing that flavonols have the ability to alleviate cognitive impairment. Although the results from animal studies are promising, clinical and epidemiological studies are still needed to advance our knowledge on the potential of flavonols to improve cognitive decline in diabetic patients.

## 1. Introduction

Diabetes mellitus (diabetes) is a group of severe metabolic diseases characterized by increased blood glucose levels resulting from a deficiency of insulin secretion, body resistance to insulin, or both [1,2,3]. Over the last few decades, the prevalence of diabetes has been growing, mainly in association with the emerging pandemic of obesity [4,5,6,7]. Among other health consequences, such as the development of heart diseases, kidney failure, vascular complications, peripheral neuropathy, and retinopathy, diabetes-associated cognitive dysfunction, also called diabetic encephalopathy, is one of the most severe outcomes of diabetes [8,9,10,11]. Regarding cognitive abilities, diabetes may increase the progression of mild cognitive impairment to dementia and Alzheimer’s disease (AD), and diabetic patients have an increased risk of developing AD [5,12,13,14].

Preclinical studies revealed that cognitive decline in diabetes results from changes in the central nervous system (CNS) that are secondary to prolonged hyperglycemia, oxidative stress, and neuroinflammation, the main drivers of neuronal dysfunction [15,16,17,18,19]. Structural and functional changes in the CNS include brain atrophy, of which hippocampal shrinkage is particularly important [20,21], impaired neurogenesis and differentiation of neural progenitor cells, reduced white matter connectivity, decreased total spine density and synaptic loss, disturbed neural transmission [22,23,24], and the appearance of electrophysiological abnormalities, including impaired long-term potentiation (LTP), altogether leading to deterioration of cognitive functions, that are observed not only in animal models but also in human patients [21,22,25].

The hippocampus, the brain region involved in learning and memory, especially in the spatial domain, appears to be highly vulnerable to hyperglycemia-induced impairments of synaptic plasticity and neuronal damage [20,26,27,28,29]. Synaptic impairments in the hippocampus are mainly caused by oxidative stress and neuroinflammation [15,19]. More importantly, increased glucose availability and enhanced accumulation of reactive oxygen species (ROS) result in increased formation of advanced glycation end products (AGEs) and upregulation of receptors for AGE (RAGE) on the neuronal and glial surfaces [30,31]. From a human perspective, these changes are more prominent in diabetic patients with mild cognitive impairment, suggesting that levels of AGEs and RAGE could be the key factors contributing to cognitive decline in humans [32,33,34].

AGEs, or Maillard products, denote a heterogeneous group of highly toxic molecules. AGEs are generated in the process of glycation, a non-enzymatic chemical transformation of amine-containing species (proteins, lipids, and nucleic acids) by reducing sugars in the blood. Although glucose displays the slowest glycation rate among natural sugars, long-term hyperglycemia and oxidative stress may profoundly accelerate AGE formation in diabetes [30]. Eventually, AGEs accumulation results in the cross-linking of intracellular and extracellular proteins, thus compromising their conformation and cellular functions and causing underlying tissue damage [35,36]. In addition, the detrimental effects of AGEs could be mediated by aberrant interactions with RAGE [37,38].

Animal studies indicate that the increased levels of AGEs and RAGE further activate the signaling pathways of mitogen-activated protein kinases (MAPKs), particularly the p38 cascade, and the transcription factor nuclear factor kappa B (NF-κB). In a vicious loop, activation of p38 and NF-κB promotes the production of more AGEs, RAGE, and ROS, therefore intensifying oxidative stress, neuroinflammation, and neurodegeneration that ultimately results in deterioration of cognitive functions and behavioral deficits [39,40,41,42].

Preclinical studies have shown that disturbances in insulin signaling also contribute to cognitive decline. As insulin is involved in neurotransmission, synaptic function and plasticity, apoptosis, and antioxidant protection, impairment of insulin signaling has detrimental consequences on brain functioning and cognition-related processes, particularly in the hippocampus [8,43,44].

Evidence from human studies suggests that dietary habits may influence the risk of developing diabetes and the overall cognitive abilities of diabetic patients [45,46,47,48]. For example, several studies have shown that a Mediterranean diet, which is rich in fruits and vegetables, may help in reducing the risk of cognitive impairment, not only in healthy individuals but in diabetic and neurological patients as well [49,50,51,52]. It is generally considered that the beneficial effects of plant nutraceuticals, such as flavonoids, are mediated by their antioxidative and anti-inflammatory properties. Moreover, a growing body of literature indicates that naturally occurring chemical constituents from fruits, vegetables, and common beverages that are capable of reducing oxidative stress and neuroinflammation may also ameliorate cognitive deficits [53,54,55]. Accordingly, dietary interventions, particularly those that are based on increased consumption of fruits and vegetables, are considered possible preventive strategies and promising approaches for slowing down the loss of cognitive abilities [14].

Among the various phytochemicals in the diet, flavonoids are appreciated as leading bioactive molecules with potentially beneficial effects on human health. However, such considerations are based predominantly on the results from in vitro and preclinical studies rather than human studies. Flavonoids not only showed neuroprotective, antioxidative, and anti-inflammatory effects but were also effective in controlling blood glucose levels, insulin secretion, and insulin sensitivity [56,57,58,59,60,61,62,63]. The beneficial effects of dietary flavonoid intake on the preservation of cognitive abilities, both in animals and humans, have also been reported [53,54,64,65,66].

## 2. Cognitive Dysfunction in Animal Models of Diabetes

Cognition encompasses mental processes such as perception, attention, learning and memory, thinking, language, and executive functions [67,68,69]. As mentioned earlier, diabetes is one of the major risk factors contributing to cognitive deterioration, along with age and some specific genetic predispositions. In diabetic patients, loss of cognitive abilities is evident in several domains. Most commonly, they have impaired psychomotor efficiency, cognitive flexibility, speed of information processing, attention, language comprehension, visuospatial abilities, and executive functions [14,15,16,70]. Moreover, cognitive impairment is usually accompanied by impaired social functioning, which altogether negatively affects the quality of patients’ lives [71].

Although clinical trials on the efficacy of flavonols are lacking, intervention studies with diets rich in fruits and vegetables provide promising evidence for their potential efficacy in alleviating cognitive decline in humans. However, a more comprehensive understanding of the cellular and molecular mechanisms underlying cognitive impairment and the effects of flavonoids has been achieved through studies in diabetic animal models. Understanding these mechanisms is pivotal, as it may guide the development of future therapeutic strategies to preserve and restore cognitive functions in diabetic patients. Therefore, this review focuses primarily on preclinical research, with sporadic references to human studies in the context of the corresponding preclinical findings to indicate their potential translational relevance.

### 2.1. The Role of AGEs and RAGEs in Diabetes-Associated Cognitive Decline

Several studies have revealed the important contribution of increased AGE formation and upregulated RAGE expression to diabetes-associated cognitive decline in diabetic animals [41,72,73,74,75]. Acting in concert, hyperglycemia and oxidative stress promote AGE generation, which in turn stimulates the production of ROS and leads to more severe oxidative stress. Thus, in addition to AGEs and RAGEs, ROS are also considered important mediators of cognitive decline in diabetes [27,76].

RAGE levels are usually low in the physiological environment, but their expression increases under various pathological conditions associated with prolonged oxidative stress and neuroinflammation, including diabetes [31,77]. In a vicious loop, increased expression of RAGE exacerbates oxidative stress, neuroinflammation, and neurodegeneration, which further promotes an increase in RAGE expression. It has been shown that RAGE levels play an important role in the development of cognitive dysfunctions, particularly those related to hippocampal spatial memory [27]. Accordingly, RAGE inhibition by pharmacological, mutational, or genetic approaches attenuates neuroinflammation, improves synaptic plasticity and synaptic function, reduces levels of apoptotic markers (Bax, caspase-3), and prevents neuronal damage and death, ultimately improving results on cognitive tasks in preclinical diabetic models [27,74,75,77].

As previously mentioned, diabetes represents a significant risk factor for AD [76,78,79], and AGEs and RAGE are considered a possible molecular link between diabetes and AD [80]. The defining histopathological hallmarks of AD are extracellular deposits of amyloid β (Aβ) and intracellular aggregates of hyperphosphorylated tau protein in neurofibrillary tangles [81,82,83]. AGEs promote the accumulation of both Aβ and tau. Namely, by activating glycogen synthase kinase-3β (GSK-3β), the main tau kinase, AGEs stimulate tau hyperphosphorylation and accumulation of neurofibrillary tangles, whereas through the activation of the AGEs/RAGE/NF-κB pathway, AGEs promote production and aggregation of Aβ [76,84,85,86,87]. In humans, it has been shown that the G82S RAGE polymorphism is strongly associated with AD development by affecting interactions between RAGE and Aβ [88,89]. Moreover, in a *db*/*db* mouse model of diabetes, FPS-ZM1, a high affinity RAGE antagonist, inhibited binding of Aβ to RAGE, prevented transport of Aβ across the blood brain barrier and its accumulation in the hippocampus, improved synaptic plasticity and LTP, and enabled better performance in cognitive tasks testing spatial and working memory, altogether indicating that RAGE inhibition could be an effective strategy for reducing cognitive loss [74].

### 2.2. Oxidative Stress in Diabetes-Associated Cognitive Decline

Oxidative stress is one of the main hyperglycemia-related factors that underlies the development of various diabetic complications at the molecular and cellular level [90,91].

Oxidative stress indicates a condition characterized by a prominent imbalance between ROS generation and elimination. ROS are eliminated by endogenous antioxidant defense systems, which include small non-enzymatic molecules, such as glutathione (GSH), and antioxidative enzymes, the most important of which are catalase, superoxide dismutase (SOD), and glutathione peroxidase (GPx). Increased production of ROS disturbs the activity of redox-sensitive signaling pathways and induces oxidative damage and structural changes in nucleic acids, proteins, and lipids. Ultimately, these changes threaten neuronal functioning, further leading to neuronal death and cognitive deterioration [92,93,94].

In diabetes, increased glucose stimulates the production of ROS and promotes oxidative stress. Hyperglycemia exacerbates the production of detrimental free radical species by several mechanisms, including (i) increased glucose metabolism through glycolysis and glucose autooxidation, (ii) enhanced activity of the polyol pathway (the pathway is involved in the regulation of GSH level), (iii) protein kinase C (PKC)-dependent activation of NADPH oxidase, (iv) increased flux of the hexosamine pathway (the pathway decreases the NADPH/NADP^+^ ratio), and (v) aforementioned production of AGEs [37,95].

Expression and/or activity of oxidative stress-related markers are altered in various tissues, including the brain, and are accompanied by reduced learning and memory abilities in diabetic animals. Commonly observed changes are increased production of ROS and NO, elevated levels of malondialdehyde (MDA), an end marker of lipid peroxidation, depleted GSH levels, and reduced activity of SOD, catalase, and GPx [96,97,98,99,100,101]. Hence, from a clinical perspective, compounds with antioxidative properties, in addition to classical antidiabetic drugs, are appreciated as a potential pharmacological intervention for slowing down the onset and progression of diabetic complications [15,102].

AGEs and RAGEs are important inducers of oxidative stress and oxidative damage in diabetes. One of the principal mechanisms of the interplay between AGEs/RAGE and ROS is related to mitochondria. Specifically, overactivation of the AGEs/RAGE axis impairs mitochondrial function. Since mitochondria are the main targets and producers of ROS, the AGEs-induced ROS increase exacerbates mitochondrial damage and further upregulates the production of ROS, disrupting mitochondrial energy metabolism and lowering ATP production. Ultimately, mitochondrial dysfunction impairs overall neuronal functioning, disturbs neural circuitry, and reduces cognitive capacity [103,104,105]. Accordingly, ultrastructural abnormalities of mitochondria, along with the presence of apoptotic markers and neurodegenerative changes, are commonly observed findings in the brains of diabetic mice [26,36,106,107]. Yet another mechanism of the AGEs-induced production of ROS is related to NADPH oxidase activation via Jun N-terminal kinase (JNK) and p38 MAPK signaling pathways [108].

Oxidative stress usually activates various kinase pathways that interfere with insulin signaling [109,110]. The phosphoinositide 3-kinase (PI3K)/Akt pathway is the main regulator of insulin signaling and glucose metabolism in pancreatic β cells, adipocytes, and muscle cells. Akt increases glucose uptake by stimulating the translocation of glucose transporters to the plasma membrane and upregulates glycogen synthesis by stimulating GSK-3 activity [111,112]. Furthermore, it has been shown that insulin regulates mitochondrial respiration through the PI3K/Akt pathway [113,114]. Reduced activation of the PI3K/Akt cascade impairs mitochondrial function, promotes oxidative stress, and causes insulin resistance. Likewise, attenuation of mitochondrial ROS production preserves sensitivity to insulin, altogether demonstrating the importance of proper insulin signaling and a balanced redox environment in cellular functioning and the prevention of diabetes-related metabolic abnormalities [109,115,116]. Akt signaling is also important for neuronal survival. Reduced pAkt/Akt ratio and increased expression of cytochrome c and caspase-3 are found in apoptotic cortical neurons of diabetic rats [117].

In addition, affecting mitochondria, oxidative stress disturbs the function of the endoplasmic reticulum (ER) and induces ER stress. In turn, ER stress exacerbates the production of free radicals, which in turn promote ER stress. In addition to ROS, increased glucose levels and disturbance of insulin signaling also contribute to ER stress and insulin resistance, at least in hippocampal neurons [15,118]. C/EBP Homology Protein (CHOP) is an important mediator of ER-induced apoptosis under hyperglycemic conditions. Its increased expression in the hippocampus of diabetic animals was accompanied by the induction of apoptosis and autophagy, as well as reduced learning and memory capacity, demonstrating the important role of ER stress in diabetes-induced neurotoxicity and cognitive impairment [21,26,98,119].

### 2.3. Neuroinflammation in Diabetes-Associated Cognitive Decline

Oxidative stress is tightly linked to neuroinflammation. The transcription factor NF-κB is the main regulator of the inflammatory response. It tunes transcriptional programs and modulates the expression of hundreds of genes involved in innate immunity. NF-κB is also an important sensor of the redox state in the cellular microenvironment [15,120]. As the enhanced production of ROS is a typical hallmark of immune cell activation, the inflammatory response and redox equilibrium are connected in a vicious cycle that drives the progression of pathological changes in various diseases, including diabetes. Increased levels of oxidative stress indicators and activation of the NF-κB cascade are concomitant findings in the brains of diabetic animals [98,100,121,122]. Accordingly, neuronal dysfunction in animal models is closely related to NF-κB activation and the consequent release of proinflammatory and prooxidative molecules, such as cytokines tumor necrosis factor (TNF)-α and interleukin (IL)-6, and ROS- and ROS-generating enzymes such as inducible nitric oxide synthase (iNOS) [97,100,123,124,125]. Ultimately, oxidative stress and neuroinflammation initiate a cascade of apoptotic events that underlie neuronal loss and reduced learning and memory capacity [98,100,124,126].

The hyperglycemia/AGEs/RAGE axis not only contributes to oxidative stress but also plays an important role in the sustained activation of NF-κB. In a feedback loop, activation of NF-κB stimulates RAGE expression and ROS generation, driving the progression of diabetic complications, including those related to cognitive abilities [27,34,127,128,129]. Accordingly, compound FPS-ZM1, a RAGE-specific inhibitor, was able to attenuate activation of NF-κB and alleviate cognitive deficits in hyperglycemic mice [27,40,130]. RAGE-mediated activation of the NF-κB pathway increases the production of several cytokines and ultimately impairs neuronal structure and functioning, particularly in the hippocampus and frontal cortex [73,74,77,120,131]. For example, in spontaneously diabetic BB/Wor rats with confirmed neurobehavioral deficits, increased expression of RAGE and stimulation of the NF-κB pathway, together with increased levels of TNF-α, IL-1β, IL-2, and IL-6, have been observed in the hippocampus [73]. Upregulation of pro-inflammatory mediators in the diabetic brain has also been reported in other preclinical models [74,101,131,132].

The p38 pathway has been identified as a critical mediator of the RAGE-induced inflammatory response due to its prominent role in the activation of NF-κB signaling [42]. High glucose levels promote p38 phosphorylation, which in turn increases phosphorylation of NF-κB subunit p65 and nuclear translocation of NF-κB, ultimately resulting in caspase-3 activation and neuronal loss in the hippocampus [42]. In streptozotocin-induced and *db*/*db* diabetic mice, hyperglycemia promotes direct binding of RAGE to mitogen-activated protein kinase kinase 3 (MKK3) and initiates assembly of the MEKK3-MKK3-p38 signaling module, which subsequently accelerates activation of the p38/NF-κB pathway. Inactivation of these two cascades reduces neuronal loss, improves synaptic plasticity in the hippocampus by modulating the function of glutamatergic AMPA receptors, and ameliorates behavioral deficits [42]. Of note, the functions of AMPA and NMDA receptors, which are involved in hippocampal LTP, are also regulated by RAGE and p38 kinase [75,133,134]. Thus, it is likely that inhibition of these pathways could have a great potential for alleviating symptoms of cognitive dysfunction in diabetic patients. In the hippocampus of streptozotocin-induced diabetic mice, the increase in RAGE expression was also accompanied by reduced JNK activity, i.e., a decreased pJNK/JNK ratio, and downregulation of the JNK kinase pMEK7, which correlated with a decrease in neuronal excitability [75]. An important role of RAGE in the activation of the p38/NF-κB pathway has also been confirmed in other cells and animal tissues affected by pathological diabetic changes [135,136,137]. In line with these findings, anti-inflammatory molecules acting as AGEs and RAGE inhibitors are considered promising candidates for pharmacological interventions in alleviating the severity of diabetic complications [31,138,139,140].

As mentioned previously, chronic hyperglycemia disturbs ER homeostasis, resulting in ER stress, irreversible unfolded protein responses, and the induction of death signaling pathways [141,142]. ER stress further leads to glucose intolerance and systemic insulin resistance; however, these changes could be prevented by NF-κB inhibition [143]. In concert with oxidative stress, ER stress promotes NF-κB activation, neuroinflammation, and neuronal apoptosis, with detrimental consequences for cognitive performance [26,98,119,144]. It seems that the hyperglycemia-induced effects on ER stress are mediated by hyperactivation of JNK signaling. In diabetic rats, increased expression of ER markers in the hippocampus was associated with increased JNK phosphorylation, NF-κB activation, and increased release of the proinflammatory cytokines TNF-α and IL-6, whereas in vitro studies demonstrated that inhibition of ER stress or JNK activity attenuates neuroinflammation induced by high glucose [144]. A study performed on primary hippocampal neurons exposed to high glucose revealed yet another role of the JNK pathway in ER stress. Inhibition of the JNK signaling pathway reduces the expression of autophagy markers, whereas autophagy inhibition exacerbates ER stress and ER stress-induced apoptosis, indicating the neuroprotective role of autophagy and the contribution of the JNK pathway in the activation of autophagy [26].

The complement system, part of the innate immune response, is also activated in diabetes [145,146]. Under physiological conditions, complement signaling plays an important role in synaptogenesis, pruning, and neuronal network plasticity [147,148,149]. In diabetes, activation of components of the complement system contributes to insulin resistance and cognitive decline, the latter likely being related to impaired synaptic plasticity [150,151]. Increased synaptic deposition of complement 3 (C3), the central component of the complement system, and the reduced density of synaptophysin, a synaptic marker, have been observed in the hippocampus of diabetic mice [40]. Of note, concentrations of the complement C3 in the blood of diabetic patients correlate with the severity of diabetes-related complications, including neuropathy [152]. For example, scores on the Digit Symbol Substitution Test were negatively correlated with serum C3 levels [28]. Furthermore, treatment with FPS-ZM1, a RAGE antagonist, attenuated the C3 increase in the brains of diabetic mice. FPS-ZM1 also prevented upregulation of p-p38 and NF-κB, suggesting that C3 induction via the RAGE/p38/NF-κB pathway could be involved in synaptic degeneration and cognitive deterioration [40]. A recent study found that overexpression of complement component 3 reduced the levels of LTP-related proteins and demonstrated an important role for astrocytic NDRG2 protein in C3 activation via the NDRG2/NF-κB/C3 cascade. Downregulation of NDRG2 expression was found to exacerbate C3 activation by promoting phosphorylation of NF-κB. Accordingly, a C3aR antagonist rescued dendritic spines and synaptic function and prevented memory loss in diabetic mice. Based on the results of the weighted gene co-expression analysis, the authors suggested that activated microglia stimulate astrocytes to produce large amounts of C3, which further amplifies the complement cascade and inflammation and disturbs synaptic plasticity and cognitive performance [28].

In addition to its well-recognized role in innate immunity, NF-κB is constitutively expressed in neurons and acts as a regulator of memory formation [153]. It is important for the conversion of short-term to long-term memory and is activated during LTP induction. Modulation of NF-κB activity therefore affects hippocampal synaptic plasticity as well as memory and spatial abilities [154,155,156]. The effect of NF-κB on memory is mediated by its downstream targets, some of which are neurotrophic factors, transcription factors, and molecules involved in neuronal outgrowth [157]. It is also involved in neuroprotection, neuronal transmission, and adult neurogenesis [158].

### 2.4. Other Factors Contributing to Cognitive Dysfunction in Diabetes-Induced Cognitive Decline

Brain-derived neurotrophic factor (BDNF) plays a crucial role in hippocampal synaptic plasticity and neuronal survival. BDNF is a growth factor, a member of the neurotrophin family, that modulates neurophysiological processes related to learning and memory storage, such as the activity of glutamatergic neurons and LTP [159]. BDNF also stimulates neuronal outgrowth and connectivity. Its effects are mediated by the activation of tropomyosin receptor kinase B (TrkB), a member of the family of tyrosine kinase receptors [159]. Most of the studies have found reduced BDNF levels, both in diabetic animals and human patients, in a correlation with the intensity of memory decline, suggesting the important contribution of BDNF in the pathophysiology of cognitive impairment [97,160,161,162,163,164,165]. Furthermore, the prevalence of depression is higher in diabetic patients, with a more prominent decrease in serum BDNF levels [166]. The relationship between fasting blood glucose and BDNF levels is not so clear-cut, as results indicating both a positive correlation [167] and no correlation [168] have been reported. A study performed on 4-week-old prediabetic *db*/*db* mice has shown that treatment with BDNF may prevent an increase in blood glucose levels, demonstrating the preventive potential of BDNF against the development of diabetes [169]. It seems that BDNF requires insulin to demonstrate the blood glucose-lowering effect. Namely, BDNF itself did not affect blood glucose levels in either control animals or streptozotocin-treated mice but increased the hypoglycemic effect of administered insulin in diabetic mice [170]. However, another study reported that the effect of BDNF is insulin-independent and related to the inhibition of glucagon secretion [171]. A study performed in humans also demonstrated a correlation between BDNF levels and impaired glucose metabolism [172]. In addition, the Val66Met (rs6265) polymorphism in the BDNF gene is likely associated with the disease progression in diabetic patients, with the Met allele acting as a protective factor by regulating dietary intake [173].

In diabetic animals, the reduced BDNF levels are accompanied by increased levels of proinflammatory cytokines, enhanced apoptosis, and neuronal loss in the hippocampus, demonstrating the important role of BDNF in neuronal survival [101]. Hence, treatment with or overexpression of BDNF could be considered a potential approach for reducing neuroinflammation in the diabetic brain and improving cognitive abilities. In mice with streptozotocin-induced diabetes, overexpression of BDNF suppressed microglial activation and production of the cytokines TNF-α and IL-6. BDNF also prevented upregulation of RAGE and attenuated activation of the NF-κB pathway, and these effects were mediated by suppression of the high mobility group protein B1 (HMGB1)/RAGE/NF-κB pathway [41]. Namely, RAGE belongs to the immunoglobulin superfamily and binds other ligands, including HMGB1, in addition to AGEs [36].

Peroxisomal proliferator-activating receptor gamma (PPAR-γ) is yet another transcription factor involved in the regulation of glucose homeostasis that plays an important role in diabetes-associated cognitive dysfunction [174]. Its levels are decreased in the brains of diabetic animals, whereas pharmacological stimulation of PPAR-γ activity promotes neurogenesis, prevents neurodegeneration, and improves cognitive abilities [175,176,177]. For example, in diabetic *db*/*db* mice, the PPAR-γ agonist rosiglitazone improved spatial and recognition memory and reduced the decrease in BDNF levels. In vitro experiments performed in hippocampal neurons also indicated that PPARγ transcriptionally upregulates BDNF expression [178]. PPAR-γ agonists improved behavioral deficits and synaptic plasticity in an animal model of AD as well [179]. PPAR-γ is also capable of modulating immune cell activation and inhibiting NF-κB, which ultimately downregulates the expression of pro-inflammatory genes and suppresses neuroinflammation [180].

A reduced expression of cAMP response element-binding protein (CREB) is also related to cognitive impairment in diabetic animals. Like PPAR-γ, CREB is a transcription factor involved in synaptic plasticity and memory. It has been shown that streptozotocin-induced hyperinsulinemia, hyperglycemia, and cognitive impairment are accompanied by decreased expression of CREB and the anti-inflammatory cytokine IL-10, whereas secretion of the proinflammatory cytokine TNF-α was found to be increased [181,182,183,184]. In alloxan-induced diabetic mice, hyperglycemia and memory impairment were likewise associated with the reduced activation of the CREB/BDNF pathway in the hippocampus, suggesting its role in long-term memory formation [183,184].

As mentioned previously, the PI3K/Akt pathway is important for proper insulin functioning. The insulin-activated PI3K/Akt signaling cascade further regulates LTP, indicating the crucial contribution of this axis to cognitive abilities [185,186]. Downstream targets of the PI3K/Akt pathway that are involved in the maintenance of glucose homeostasis include the mechanistic target of rapamycin (mTOR) (regulates protein synthesis), GSK-3β (regulates glycogen synthesis), Fox1 (regulates gluconeogenic genes), and AS160 (participates in glucose transport). It has been shown that levels of PI3K, pAkt, GSK-3β, Fox1, and mTOR are reduced in the brains of streptozotocin-induced diabetic male Wistar rats [97]. Similarly, in rats with co-morbidities of diabetes and depression, the PI3K/Akt/mTOR pathway-mediated hippocampal degeneration and increase in autophagy have been assigned to cognitive impairment [187]. Accordingly, there is accumulating evidence that the beneficial effects of various compounds on the diabetes-induced cognitive deficits are due to a re-established activation of the PI3K/Akt/mTOR pathway and reduced oxidative stress, neuroinflammation, and autophagy [188,189,190,191,192].

**Figure 1:**
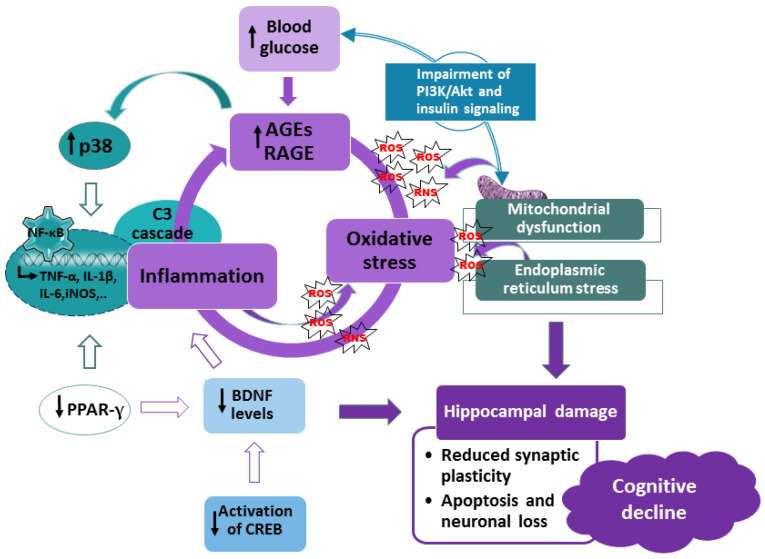
The main neuropathological mechanisms contributing to cognitive deterioration in preclinical models of diabetes.

The main pathological mechanisms contributing to cognitive decline in animal models of diabetes are summarized in Figure 1.

## 3. Methodological Approaches for Assessing Cognitive Functioning in Animal Models of Diabetes

The most common animal model of diabetes is based on streptozotocin administration. Biochemical and functional alterations induced by streptozotocin best resemble those observed in human patients. Depending on the dose and duration, both insulin-dependent (type 1, caused by destruction of β cells) and insulin-independent (type 2, caused by insulin resistance) diabetes can be induced [95]. Via glucose transporter 2 (GLUT2), streptozotocin is preferentially taken up into β cells where it has toxic effects. GLUT2 is a high-affinity transporter abundant in the pancreatic cells of rodents [193]. However, expression of GLUT2 is very low in humans, making them resistant to the diabetogenic activity of streptozotocin [194,195]. Structurally, streptozotocin is an analogue of n-acetyl glucosamine with glucose attached to nitrosourea. This nitrosourea moiety mediates the toxic effects of streptozotocin via several mechanisms, including increased production of ROS and induction of nitrosative and oxidative stress, reduced activity of the antioxidant enzymes, NO-mediated aconitase inhibition and subsequent DNA alkylation, mitochondrial dysfunction, DNA methylation, and depletion of ATP levels. These processes ultimately inhibit insulin secretion and result in diabetes [95,195]. Streptozotocin also stimulates broad infiltration of macrophages in various organs, induces systemic inflammation, increases the number of microglial cells, and elicits an inflammatory response and the production of pro-inflammatory cytokines in the brain [125,196,197].

Alloxan, a glucose analogue with hydrophilic properties, is also used as a diabetogenic agent for inducing type 1 diabetes. It can be administered intraperitoneally, intravenously, and subcutaneously in single or multiple doses; the most common option is a single intraperitoneal administration [198]. Like streptozotocin, it crosses the plasma membrane via GLUT2 and preferentially accumulates in pancreatic β cells. There are two main pathological mechanisms of alloxan action in rodents. Firstly, it inhibits glucose-stimulated insulin secretion by inhibiting glucokinase, a glucose-sensing enzyme. Secondly, it promotes ROS production via redox cycling and induces necrosis of β cells [198,199]. The main limitation of the model and the disadvantage compared to streptozotocin are that alloxan-induced hyperglycemia is relatively unstable and unpredictable, which may jeopardize the screening of the antidiabetic potential of a test drug. Unlike streptozotocin, alloxan also has a high mortality rate and induces severe damage to other GLUT2-expressing cells [200].

Genetic animal models are also being employed to study the antidiabetic effects of compounds of interest. The most widely exploited models are leptin-null mice (*ob*/*ob*) and leptin receptor mutant mice (*db*/*db*). *db*/*db* mice are a well-accepted model for type 2 diabetes, showing insulin resistance and progressive hyperglycemia and dysfunction of β cells during aging, together with the hippocampal inflammation, proteomic alterations, and behavioral and cognitive abnormalities seen in human patients [201,202]. Goto–Kakizaki (GK) rats are rats born with a reduced number of β-cells and represent a model for type 2 diabetes. They have been developed by the selective breeding of offspring with the most prominent hyperglycemic traits [203,204].

Behavioral Tests Used for the Assessment of Cognitive Abilities in Rodents

Preclinical studies investigating cognitive functions in animal models of diabetes rely on various behavioral tests developed to assess cognitive functioning in rodents, focusing primarily on learning and memory. The Morris water maze (MWM) is a widely used behavioral test to assess spatial learning and memory in rodents. Typically, animals are trained to find a hidden platform (placed in the target quadrant) in a water-filled maze, and the time it takes them to locate the platform, known as the latency period, is used as an indicator of their learning success. Subsequent test trials assess memory retention by evaluating how well animals remember the platform’s location [21,42,74,205]. The passive avoidance task assesses memory acquisition and retention using a two-chambered apparatus, with one chamber delivering a mild foot shock. During the acquisition phase, animals learn to avoid the chamber associated with the shock, and memory retention is tested in the probe trial performed after a specific delay period [86,130,177]. The elevated plus maze, which was originally developed to assess anxiety in rodents, is used to test long-term spatial memory. Here, animals are repeatedly exposed to the open arms of the maze, and the time it takes them to move from an open to a closed arm, known as transfer latency, serves as an indicator of memory consolidation and retention [206,207]. The novel object recognition test assesses aspects of declarative memory by exploiting rodents’ natural curiosity for novelty. It measures an animal’s ability to recognize and remember novel objects by using the discrimination ratio, a measure based on the time the animal spends exploring novel objects compared to previously encountered objects [178,179,208]. The Y-maze test is used to assess working and short-term spatial memory in rodents. Animals are trained to enter and remain in the non-shocking arm of the three-arm maze, and memory performance is assessed by measuring the latency to enter the safe arm and the number of correct entries during the testing trials [28,74,178,179]. The open-field test is often used to rule out motor impairments and the influence of anxiety [27,42,74].

## 4. Effects of Flavonols on Cognitive Functions in Diabetic Animals

As emphasized previously, foods rich in flavonoids may have beneficial effects on cognitive functioning [14,49]. Flavonoids comprise a large group of secondary plant metabolites with well-documented antioxidative, anti-inflammatory, and neuroprotective effects, as well as antidiabetic and memory-enhancing properties [14,53,209,210,211]. Structurally, all flavonoids have benzene and phenyl rings (rings A and B), which are bridged by the heterocyclic pyrene ring C. Based on the diversity of chemical structure (degree of oxidation and saturation, pattern of attached groups, and position at which the B-ring is connected to the C-ring), they are categorized into several subclasses [211,212]. The most important subtypes of flavonoids are flavonols, flavanols, flavones, flavanones, anthocyanins, and isoflavones [212,213]. However, to exclude the possibility that different classes of flavonoids could have slightly different effects and mechanisms of action on diabetes-related cognitive complications, we have focused on flavonols in this literature review.

The main dietary sources of flavonoids are fruits and vegetables, tea, and red wine [214,215]. Accordingly, there is great interest in the potential health-promoting effects of a diet enriched with fruit and vegetables. The Mediterranean diet is characterized by a high consumption of fruits, vegetables, and beverages rich in flavonoids, and this type of diet has been repeatedly associated with a lower risk of chronic diseases, including diabetes [216,217,218]. The composition of the various flavonoids in different foods can be assessed using the available databases on flavonoid content. However, the estimated dietary intake of flavonoids varies widely between studies. The estimation of the total dietary intake of flavonoids depends on the number of foods considered, the geographical and seasonal origin of the foods, the environmental conditions, agricultural practices, the degree of ripeness, the storage and processing of the foods, the analytical methods used for quantification, as well as the population studied, as intake may vary according to age, gender, dietary habits, income level, and ethnicity [214,215,219]. In two US studies, the total daily intake of flavonoids ranged from 189.7 mg/day to 251 mg/day. Apart from flavan-3-ols, which accounted for about 80% of the total flavonoid intake, flavonols were the second most abundant flavonoids in these studies. Their content is estimated at 6.8% and 8% of all flavonoids [214,215]. However, in a Greek plant-based weekly menu, the daily intake of flavonoids was estimated at 118.6 mg, of which flavonols accounted for 22% [220]. Flavonols are mainly found in onions, kale, broccoli, apples, cherries, berries, tea, and red wine. The most abundant flavonols in the diet are quercetin, kaempferol, and myricetin [219,220]. Structurally, flavonols have an oxo group at position 4, a hydroxyl group at position 3, and a 2,3-double bond on ring C, which is particularly relevant in the context of their redox properties and health-promoting effects [62]. Flavonols are one of the most studied flavonoids. Their great potential against various chronic diseases has been demonstrated in many studies [62,211,221]. Moreover, it has been suggested that the health-promoting effects of flavonols are superior to those of other subclasses of flavonoids due to their complex mechanisms of action [221].

Based on numerous preclinical studies, restoration of redox homeostasis is appreciated as a promising therapeutic option to manage cognitive impairment in diabetes. Flavonols in general possess remarkable antioxidant abilities. They exert antioxidant effects via several mechanisms, including (i) direct ROS scavenging, (ii) induction of the endogenous mechanisms of antioxidative defense, (iii) chelation of metal ions that otherwise may initiate ROS formation via Fenton chemistry, and (iv) regulation of aberrant redox-sensitive signaling pathways [211,222,223,224]. In humans, plasma levels of antioxidants and total antioxidant status are positively correlated with the general intake of fruits and vegetables [225,226,227,228]. Evidence is growing that increased consumption of foods rich in antioxidants, or supplementation with antioxidants, may reduce levels of oxidative stress markers [229] and even improve cognitive performance in various conditions, both in humans and animals [230,231].

In addition, possessing powerful antioxidant abilities, flavonols are capable of regulating oxidative and inflammatory signaling cascades involved in neuronal survival. They also target various proteins important for cognitive processes, such as BDNF, thus preserving the structural integrity and functionality of neural circuits, which is ultimately beneficial for overall cognitive performance [206,211,224,231]. As will be shown later, the results of many preclinical studies indicate that individual flavonols are effective against cognitive decline in diabetes. On the contrary, there are only a few studies investigating the effects of flavonols and foods rich in flavonols on diabetic patients. In a longitudinal study that lasted for more than 6 years and was performed on more than 10,000 middle-aged participants (45–64 years), dietary intake of food enriched with flavonols slowed down cognitive decline over time [232]. By using the combined change score from the delayed word recall test, the Wechsler Adult Intelligence Scale-Revised (WAIS-R) digit symbol subtest, and the word fluency test, it was found that intake of flavonols, particularly myricetin, kaempferol, and quercetin, positively correlates with the preservation of cognitive abilities [232]. The effect was the most pronounced for the digit symbol subtest; in the word recall test, it was not significant and was marginal in the word fluency test. The association between a diet rich in flavonols and the slower progression of cognitive disabilities was also demonstrated in an American study. This study was performed on almost 1000 participants aged 60–100 years whose cognitive abilities were evaluated with a battery of 19 cognitive tests. The obtained results revealed better global cognition, working memory, episodic and semantic memory, and perceptual speed in participants with a higher dietary intake of flavonols, particularly quercetin and kaempferol [233]. Regarding diabetes, in a study lasting for app. 12 years that included almost 3000 individuals, the intake of flavonols and flavan-3-ol reduced the incidence of diabetes by 26% and 11%, respectively. Associations between the intake of flavonoids from other classes (flavones, flavanones, anthocyanins, and polymeric flavonoids) and diabetes risk were not found [56].

### 4.1. Mechanisms Underlying the Beneficial Effects of Quercetin in Diabetic Animals

Quercetin (3,3′,4′,5,7-pentahydroxyflavone) is one of the most widely distributed flavonols in the human diet. In large amounts, it can be found in apples, onions, broccoli, berries, kale, green tea, red wine, seeds, and nuts [234]. It possesses powerful antioxidant and anti-inflammatory abilities [210,211,235] that underlie its antidiabetic effects. Based on a 100-item food frequency questionnaire, daily quercetin intake in a Chinese population has been estimated to be 20.9 ± 2.32 mg/day. More importantly, dietary quercetin consumption negatively correlated with diabetes prevalence, suggesting the protective role of quercetin in the development of the disease [236].

The effects of quercetin on memory dysfunction have been documented in animal models of streptozotocin-induced diabetes. In the MWM test, diabetic rats exhibited higher escape latency during training trials and reduced time spent in the target quadrant in the probe trial compared to control animals, whereas in the elevated plus maze task, diabetic animals showed increased latency. Upon diabetes induction, the changes observed in the MWM and the elevated plus maze tasks were reversed in rats receiving quercetin (5, 10, and 20 mg/kg, twice daily for 30 days) [206]. In another experiment in the same study, rats received 20 and 40 mg/kg quercetin (twice daily) during training trials (from days 31 to 35). Acute treatment with the higher dose reduced the escape latency and increased the time spent in the quadrant with a platform [206], indicating that even the short-term administration of quercetin may improve cognitive performance.

Numerous preclinical studies have shown that quercetin reduces blood glucose levels in diabetic animals [237,238,239,240] and increases sensitivity to insulin [241,242]. Accordingly, it has been suggested that the beneficial effects of quercetin on the improvement of cognitive functions at least partially rely on its anti-hyperglycemic properties, preventing the hyperglycemia-mediated effects on the induction of oxidative stress and neuroinflammation via the AGEs/RAGE axis [206,243]. The restoration of glucose metabolism is usually accompanied by the prominent antioxidative effects of quercetin at the systemic level and in specific tissues, particularly in the pancreas and liver. Thus, quercetin administration reduced the extent of lipid peroxidation, improved the activities of antioxidative enzymes, mainly SOD, catalase, and GPx, and restored levels of small antioxidants such as GSH and vitamins C and E [237,244,245,246,247,248]. Likewise, quercetin-mediated effects on cognitive functions were associated with improved activity of antioxidative enzymes and reduced amounts of oxidative stress markers [249,250].

In addition to the mentioned activities, quercetin acts as an acetylcholinesterase (AChE) inhibitor, which very likely contributes to its memory-enhancing abilities [209,243,249]. Quercetin-mediated attenuation of cholinergic dysfunction has been demonstrated in the hippocampus and cerebral cortex of streptozotocin-induced diabetic rats (quercetin was applied at doses of 25 and 50 mg/kg for 40 days) [249]. In addition, quercetin reduced the MDA levels in a dose- and brain region-specific manner and prevented impairment of enzymes involved in the regulation of purinergic transmission, at least in the synaptosomes from the cerebral cortex [249]. Likewise, in mice administered with streptozotocin, quercetin (2.5, 5, and 10 mg/kg, p.o. for 21 days) reduced the increase in AChE activity, decreased MDA and nitrite levels, and increased GSH content [243]. In addition, attenuating oxidative stress and cholinergic dysfunction, quercetin restored cerebral blood flow and ATP levels and prevented memory impairment. Effects of quercetin on streptozotocin-induced memory impairment were evaluated in the MWM test (quercetin at doses 5 and 10 mg/kg decreased the mean latency time after the third session) and the passive avoidance test (quercetin increased the transfer latency time in retention trials in comparison with acquisition trials) [243].

Another study has shown that the mechanism underlying the beneficial effect of quercetin on cognitive performance is mediated by the increased activation of deacetylase sirtuin 1 (SIRT1) and inhibition of ER stress. Namely, among other functions, SIRT1 regulates insulin secretion and blood glucose levels and may have an inhibitory effect on signaling pathways implicated in ER stress and cognitive dysfunction. In female *db*/*db* mice, quercetin at a dose of 70 mg/kg (applied for 12 weeks) attenuated glucose tolerance and insulin resistance and improved cognitive performance. It shortened the time needed to find the hidden platform in the MWM test, and following platform removal, it increased the time spent in the target quadrant and time engaged in the exploration of the platform area [250]. Likewise, quercetin improved performance in the novel object recognition test. Together with these effects, quercetin upregulated SIRT expression as well as the expression of synapse-related proteins and neurotrophic factors, including BDNF and NGF, and reduced the expression of ER stress markers. Quercetin-mediated improvement of cognitive abilities was also accompanied by reduced oxidative stress, reduced expression of proapoptotic proteins, and attenuated neuronal apoptosis and neurodegeneration in the hippocampal and cortical areas, altogether suggesting the promising potential of quercetin for relieving symptoms of diabetes-induced cognitive decline [250]. A similar study that was performed in streptozotocin-induced diabetic rats also demonstrated the ability of quercetin (100 mg/kg b.w., applied orally in aqueous solution for 15 days) to reduce serum glucose levels and increase SIRT1 expression [238].

In addition to central effects, quercetin also restored activation of the adenosine 5′-monophosphate (AMP) activated protein kinase (AMPK)/peroxisome proliferator-activated receptor-γ coactivator 1α (PGC-1α) pathway and prevented mitochondrial dysfunction in an animal model of diabetic peripheral neuropathy [246]. The hyperglycemia-induced impairment of mitochondrial function has been suggested as one of the major pathological mechanisms of diabetic neuropathy [246]. This chronic microvascular complication of diabetes results in neuropathic pain due to the activation of purinergic P2X_4_ receptors in the dorsal root ganglia [251]. In the streptozotocin-induced rats, oral administration of quercetin (for 6 weeks at doses of 30 and 60 mg/kg/daily) improved the paw withdrawal threshold (an indicator of mechanical allodynia), the motor nerve conduction velocity, ultrastructural abnormalities of sciatic nerves, loss and morphological changes of neurons in the dorsal root ganglion, axonopathy, expression of myelin proteins, and demyelination of myelin sheet [246]. Together with the alleviation of mitochondrial degeneration and restoration of ATP production, quercetin increased expression of phosphorylated AMPK, PGC-1α, SIRT1, nuclear respiratory factor 1 (NRF1), and mitochondrial transcriptional factor A (TFAM), suggesting that its neuroprotective effects are closely related to the restoration of mitochondrial energy metabolism and activation of the AMPK/PGC-1α pathway. In general, the energy status of cells and mitochondrial biogenesis are controlled through the AMPK/PGC-1α pathway, and upregulation of its activity usually preserves mitochondrial function in hyperglycemic conditions [246]. Furthermore, in dorsal root ganglion neurons exposed to high glucose, quercetin activated the Nrf2 pathway and inhibited the NF-κB signaling cascade, suppressing the production of proinflammatory cytokines and the expression of iNOS [252]. Of note, the SIRT1/PGC-1α axis also participates in the regulation of redox homeostasis in mitochondria, and its stimulation may increase the ROS-detoxifying abilities, which is in line with the observed antioxidative effect of quercetin [246,252]. In addition, quercetin may prevent upregulation of the P2X_4_ receptors and activity of the p38 pathway in diabetic rats [253], whereby the latter is known to be upregulated in the diabetic brain and likely contributes to cognitive dysfunction [42,254].

The anti-inflammatory effects of quercetin also play an important role in rescuing cognitive abilities. It was shown that pure quercetin and quercetin-conjugated superparamagnetic iron oxide nanoparticles (applied at 25 mg/kg for a period of 35 consecutive days) may attenuate expression of microRNA-146a (miR-146a), an inflammation-sensitive microRNA, and expression of NF-κB and NF-κB-related downstream targets, such as TNF-α, in the hippocampus of the streptozotocin-induced diabetic rats. Molecular docking revealed that the inhibitory effect of quercetin on the NF-κB pathway could be mediated by targeting the IKK protein [124]. Quercetin and quercetin-conjugated superparamagnetic iron oxide nanoparticles also alleviated diabetes-induced overexpression of redox-sensitive miR-27a, which subsequently increased levels of Nrf2 and its target genes SOD and catalase, together with the prevention of memory dysfunction [255]. A study performed with *db*/*db* mice has shown that quercetin (35 and 70 mg/kg for 12 weeks) may improve cognitive performance by regulating the Sirt1/nucleotide-binding domain-like receptor protein 3 (NLRP3) pathway. In particular, quercetin increased the expression of Sirt1, which is a negative regulator of the inflammatory response, and consequently reduced the expression of inflammation-related proteins, including NLRP3 and the pro-inflammatory cytokines IL-1β and IL-18, thus inhibiting the NLRP3 inflammasome activation and further neurodegenerative processes. Furthermore, quercetin enhanced the expression of synapse-related proteins and neurotrophic factors BDNF and NGF, demonstrating the co-existence of multiple mechanisms that likely function together in the restoration of cognitive abilities [256]. The anti-inflammatory potential of quercetin has also been observed in other tissues of diabetic animals and is suggested to have an important role in the antidiabetic effects of quercetin [248].

Except in rodents, the antidiabetic effects of quercetin were demonstrated in the streptozotocin-induced Arbor Acre broilers supplemented with 0.02–0.06% quercetin and were mainly attributed to the antioxidant mechanism of quercetin action. In chickens, quercetin in a dose-dependent manner improved fasting blood glucose and insulin levels, activities of antioxidant enzymes catalase and SOD, MDA and nitric oxide content in serum and liver tissue, and regulated expression of genes related to the PI3K/Akt pathway [257].

As mentioned, besides pure quercetin, administration of quercetin-conjugated superparamagnetic iron oxide nanoparticles demonstrated a positive effect on learning and memory dysfunction in streptozotocin-induced diabetic animals. These nanoparticles were used to investigate the possibility of overcoming the high clinical doses of quercetin due to its limited permeability across the blood-brain barrier, low bioavailability, and rapid metabolic changes in the gastrointestinal system. In diabetic rats, nanoparticles demonstrated better efficacy in the improvement of cognitive abilities in comparison to free quercetin (both were applied at a dose of 25 mg/kg for 35 days). In the MWM test, rats that received quercetin-conjugated superparamagnetic iron oxide nanoparticles showed reduced escape latencies in the repeated training trials compared to diabetic animals and the free quercetin group, the shortest swimming path length before finding the hidden platform in subsequent trial days, and spent the highest amount of time in the target quadrant. Likewise, free and conjugated quercetin increased the step-through latency in the passive avoidance task, indicating the beneficial effect of quercetin on the retrieval of the fear memory. Treatment with quercetin also prevented the apoptotic cell death of granular neurons in the hippocampus [239]. As the side-effects of nanoparticles were not noticed, and considering that quercetin in the conjugated form was more effective in improving memory performances compared to free quercetin, it is likely that novel therapeutic strategies for oral quercetin delivery in managing cognitive decline in diabetes should be oriented towards the development of conjugated formulations and better characterization of their mode of action, stability in the circulation, brain distribution, and safety profile.

The anti-diabetic effects of quercetin have also been investigated in humans. Ostadmohammadi and co-authors performed a meta-analysis of randomized controlled trials, concluding that supplementation for a period longer than 8 weeks at doses higher than 500 mg/day may reduce fasting plasma glucose [258]. In patients with type 2 diabetes, aged 30–60 years, daily oral intake of lower doses of quercetin (250 mg/day, every 2 weeks for 8 weeks) was sufficient to increase the total antioxidant status and antioxidative defense, although this dose was insufficient to regulate glycemic parameters (fasting blood glucose and serum insulin) [259]. Studies focused on cognitive abilities following the quercetin supplementation protocol are not numerous. In a randomized, double-blind, placebo-controlled clinical trial including 70 Japanese men and women aged 60–80 years, intake of quercetin-rich onion (the daily quercetin intake was estimated to be 50 mg as aglycone equivalent) for 24 weeks improved the Mini-Mental State Examination scores. Although the mechanism behind the quercetin action was not elucidated, the authors suggested that quercetin reduces cognitive decline by improving emotional functions and motivation [260]. We have found no study investigating the effect of quercetin supplementation on cognitive functions in diabetic patients, indicating that such clinical trials are urgently warranted for the evaluation of the cognitive-enhancing potential of quercetin in the human population.

### 4.2. Effects of Other Flavonols on Diabetes-Induced Cognitive Decline in Animal Models

In contrast to quercetin, whose effects are relatively well-studied, the potential of other flavonols against cognitive impairment in diabetes has yet to be explored. Although there are studies that investigate the antidiabetic effects of other flavonols in different tissues, studies demonstrating their effects on the brain and cognitive abilities in preclinical diabetic settings are not numerous.

Rutin (quercetin-3-O-rutinoside) is flavonol with the attached disaccharide rutinose on the quercetin backbone. In various neuropathological conditions, rutin displayed antioxidative, anti-inflammatory, and neuroprotective effects [261,262,263].

Protective and regulatory effects of rutin based on antioxidative and anti-inflammatory mechanisms have been demonstrated in non-brain tissues of diabetic (streptozotocin-induced and *db*/*db*) animals [264,265,266,267,268,269,270]. Thus, rutin reduced blood glucose level [264,265,266], increased insulin secretion and glycogen storage [264,266], improved lipid profile and biochemical parameters [267,269], reduced generation of AGEs [270], regulated the PI3K/Akt/GSK-3β pathway [270], prevented degenerative changes [264,267,268], reduced the MDA levels [268], increased the GSH content and non-enzymatic and enzymatic antioxidative defense [264,265,266,268], and attenuated production of proinflammatory cytokines TNF-α and IL-6 [269].

In the brain of the streptozotocin-induced rats, rutin (applied for 45 days at a dose of 100 mg/kg) improved the antioxidant status by increasing the non-enzymatic (GSH) and enzymatic antioxidant protection (SOD, catalase, GPx, glutathione reductase) and preventing morphological changes [266]. Effects of rutin on cognitive performance have been investigated in the intracerebroventricular-streptozotocin (ICV-STZ)-infused rats, a model of sporadic AD that overlaps in many symptoms with diabetes-induced pathological changes, such as learning and memory deficits, impaired glucose metabolism, oxidative stress, and neuroinflammation. Pretreatment for 3 weeks with rutin (25 mg/kg) attenuated oxidative stress, as evidenced by the reduced lipid peroxidation and nitrite accumulation and the increased GSH content and activity of the GPx, glutathione reductase, and catalase in the hippocampus. In the same study, rutin demonstrated remarkable anti-inflammatory activity by suppressing microglial activation and expression of iNOS and NF-κB, which likely contributed to the preservation of hippocampal morphology and improved performance in the MWM task [271]. Regarding diabetes, the effects of rutin have been studied in diabetic retinas. Administration of rutin (100 mg/kg) for 5 weeks increased levels of BDNF and NGF, increased GSH levels, attenuated lipid peroxidation, and demonstrated an antiapoptotic effect by decreasing levels of caspase-3 and upregulating expression of Bcl-2 [272]. This may suggest that similar protective mechanisms may be effective against diabetes-induced cognitive complications in the brain.

Troxerutin is a semi-synthetic flavonol derived from rutin that has powerful antioxidant properties. Much evidence suggests that it may improve cognitive decline in animals treated with streptozotocin. In the hippocampus of diabetic rats, troxerutin (150 mg/kg/day for 6 weeks, starting 4 weeks after streptozotocin administration) reduced lipid peroxidation and increased SOD activity, attenuated gene expression of the NADPH oxidase subunits (NADPH oxidase is an important source of ROS), stimulated the nuclear translocation of Nrf2, and increased the cytosolic fraction of the antioxidant enzymes heme oxygenase-1 (HO-1) and NAD(P)H:quinone oxidoreductase (NQO1). Although troxerutin did not regulate blood glucose levels at this dose, the memory-enhancing effect of troxerutin was confirmed in the MWM test and mainly attributed to the antioxidant mechanisms of its action. Taken together, these results suggest that the effects of troxerutin on improving spatial memory were mediated through the regulation of the Nrf2/ARE pathway and suppression of oxidative stress [273]. In rats treated for 6 weeks, but starting at 12 weeks after streptozotocin administration, troxerutin also improved cognitive performance in the MWM test, regulated oxidative stress-related parameters (SOD activity, GSH, and MDA levels), and stimulated expression of the catalytic and modifier subunits of the glutamate cysteine ligase that catalyze the rate-limiting step of GSH synthesis [274]. Similar results were shown when troxerutin was used in the early stages of diabetes, which resembles prophylactic use. Rats were injected with 60 mg/kg of troxerutin for 12 weeks, starting 72 h after streptozotocin injection. This type of treatment improved learning and memory abilities, increased Nrf2 expression in the hippocampus, stimulated SOD activity, and decreased lipid peroxidation. In addition, it preserved normal hippocampal morphology, altogether indicating that troxerutin is effective in delaying cognitive decline when used as a preventive intervention [275]. The contribution of the anti-inflammatory mechanisms to the memory-enhancing effects of troxerutin has also been revealed. In the streptozotocin-induced diabetic rats, troxerutin (administered at 150 mg/kg for 1 month, from 7th to 10th weeks after streptozotocin injection) inhibited expression of NF-κB and its adaptor proteins TRAF-6 and IRAK-1, most likely by targeting regulatory miR-146a [276]. In high-fat diet-fed rats, troxerutin attenuated the neuroinflammatory response (as evidenced by the decreased production of proinflammatory cytokines) and increased BDNF levels, which may be beneficial in diabetic conditions as well [277]. Furthermore, in AD preclinical models, troxerutin inhibited AChE activity and stimulated the PI3K/Akt pathway, demonstrating its ability to target the key molecular mechanisms underlying the cognitive dysfunction in diabetes [278].

Like quercetin, myricetin (3,3′,4′,5,5′,7-hexahydroxylflavone) is a flavonoid from the flavonol class that is present in various fruits and vegetables, nuts, berries, and beverages [279]. Animal and clinical studies revealed its hypoglycemic effect and ability to increase the sensitivity to insulin and enhance glycogen synthesis and glucose utilization, together with the prominent antioxidative and anti-inflammatory activities that are most likely essential for its protective effects against diabetic complications in various tissues [280,281,282,283,284,285]. Myricetin possesses five hydroxyl groups that underlie its potent antioxidative activity. On the other hand, the glucoregulatory activity of myricetin is probably based on its ability to act as an agonist of the glucagon-like peptide 1 (GLP-1) receptor. Briefly, GLP-1 stimulates insulin secretion and regulates blood glucose levels but shows extremely low stability. Hence, other natural or synthetic GLP-1 receptor agonists are suggested as a more promising therapeutic approach in diabetes. In that regard, it is considered that molecular modeling and chemical modifications of myricetin molecules could greatly advance a search for more potent GLP-1 receptor agonists for further clinical evaluation [286]. In the streptozotocin-induced diabetic rats lacking insulin, it was suggested that the acute administration of myricetin increases the release of β-endorphin and lowers plasma glucose levels by activating the opioid μ receptors in peripheral tissues [287]. In diabetic peripheral neuropathy, myricetin (0.5–2.0 mg/kg/day, injected intraperitoneally for 2 weeks from the 21st day after streptozotocin administration) was able to reduce abnormal sensations and improve nerve morphology and conduction velocity, as well as the blood flow. More importantly, myricetin reduced the generation of AGEs and ROS, increased the activity of the antioxidant enzymes, activated the Nrf2 pathway, and improved the antioxidant defense, suggesting its potential therapeutic value for other diabetic complications as well [288]. In addition, myricetin inhibited AChE activity [209] and enhanced memory functions by protecting hippocampal neurons in a rat model of AD, which may suggest its potential against diabetes-induced cognitive decline [289]. Similarly, in the senescence-accelerated mouse-prone 8 (SAMP8) mice displaying accelerated aging, intake of myricetin improved performance in the novel object recognition test and the Y-maze test and upregulated expression of BDNF and NGF [290]. However, regarding the safety issues of myricetin supplementation, detailed studies should be carried out. Specifically, elevated levels of systemic and hippocampal copper are found in patients with diabetes, together with the positive correlation between serum levels of copper and deregulation of glycemic control [291,292]. On the other hand, in certain environmental conditions, such as the presence of high levels of copper, myricetin demonstrated prooxidative and cytotoxic effects [293], which theoretically may negatively affect cognitive abilities.

Dihydromyricetin is a flavonoid with great structural similarity to myricetin, although it is not classified as a flavonol (2,3-double bond of myricetin is hydrogenated in dihydromyricetin). In a diabetic mouse model (mice were fed a high-sugar and high-fat diet for 8 weeks and then administered a low dose of streptozotocin), dihydromyricetin improved spatial learning and working memory, likely by suppressing oxidative stress (i.e., it decreased MDA accumulation and increased activity of SOD, catalase, and GPx) and increasing BDNF levels, which together exerted neuroprotective effects in the hippocampus [294].

Myricitrin, a 3-O-rhamnoside of myricetin, belongs to flavonols and can be found in large amounts in the root bark of *Myrica cerifera* and *Myrica esculenta* [295]. In the streptozotocin-nicotinamide model of type 2 diabetes, the encapsulated myricitrin restored blood glucose and insulin levels and improved the total antioxidant capacity and levels of MDA, antioxidant enzymes (SOD, catalase), and apoptotic markers in the pancreas [296]. Similarly, in the high-fat diet and streptozotocin-induced diabetic mice, myricitrin decreased fasting blood glucose and attenuated gene expression of the proinflammatory cytokines in the liver [297], which suggests the antidiabetic potential of myricetin, but its effects on the brain and cognitive abilities still need to be investigated.

Kaempferol is yet another flavonol abundantly present in the human diet. It can be found in various fruits, vegetables, and beverages, such as broccoli, beans, apples, and strawberries [298,299]. In various neurological conditions, including the experimental model of sporadic AD, kaempferol regulated the NF-κB, p38, and Akt signaling cascades, attenuated ROS production, stimulated the activity of endogenous antioxidants, and increased BDNF levels. Thus, the neuroprotective effects were largely mediated by its remarkable antioxidative and anti-inflammatory activities and ability to modulate intracellular signaling pathways [300,301]. The beneficial effects of kaempferol that were based on antioxidative, anti-inflammatory, and signaling-related mechanisms have also been observed in diabetes and diabetes-induced complications [302,303,304,305,306,307]. In addition, restoring glucose and insulin levels [302,303,304,307], kaempferol suppressed activation of the AGEs/RAGE axis [304], attenuated lipid peroxidation [303,304,305,307], improved effectiveness of the enzymatic and non-enzymatic systems of antioxidative defense [303,304,305,307], reduced ROS levels [305,307], and stimulated Nrf2 activity [305,307] in the plasma and various organs of the streptozotocin-induced diabetic animals. Furthermore, kaempferol suppressed transactivation of the NF-κB [304,307] and attenuated release of proinflammatory cytokines such as TNF-α and IL-6 [304,305,307], inhibited apoptosis [304,305,306,307], regulated expression of the autophagy-related proteins and activity of the AMPK/mTOR pathway [306], restored activity of the p38 and JNK pathways [304,307] and upregulated deacetylase activity and levels of SIRT1 [305]. Regarding complications affecting neuronal cells, the effects of kaempferol were studied in streptozotocin-induced diabetic neuropathy. Kaempferol partially reduced the nociceptive pain and increased motor nerve conduction velocity by attenuating AGE formation, oxidative and nitrosative stress, and the inflammatory response [308,309]. Although, to the best of our knowledge, the effects of kaempferol on diabetes-induced cognitive decline have not yet been investigated, the results obtained for diabetic neuropathy suggest that the mechanisms providing protection in other tissues are also present in neurons and capable of improving performance in specific behavioral tests.

Fisetin is a flavonol that has shown antinociceptive effects against diabetic neuropathic pain. It reduced thermal hyperalgesia and mechanical allodynia and demonstrated prominent antioxidative activity (based on targeting the Nrf2 pathway) and the ability to attenuate the inflammatory response (based on regulation of NF-κB activity) [310,311]. In diabetic rats, fisetin also regulated glucose levels, reduced expression of the NF-kB p65 unit (in the pancreas), and levels of IL-1β and NO in the blood [312]. The promising potential of fisetin in alleviating cognitive dysfunction was shown in old SAMP8 mice (a model for sporadic AD). In these mice, fisetin improved cognitive impairment by targeting specific proteins involved in synaptic function, cellular response to stress, brain inflammation, and modulation of the stress-activated protein kinase (SAPK)/JNK pathway [313]. All these mechanisms of action may also be relevant for improving cognitive impairment in diabetes, but further studies are needed to determine if fisetin may enhance cognitive functions in diabetic conditions.

Flavonol morin has shown neuroprotective effects in diabetic neuropathy, as evidenced by the improvement of motor and sensory nerve conduction velocities and nerve blood flow. Based on the results obtained in vitro, the authors proposed the regulatory role of morin along the Nrf2 and NF-κB signaling pathways [314]. Another study also suggested the therapeutic potential of morin in diabetic neuropathy based on the reduced cytokine levels and lipid peroxidation, increased levels of NGF and GSH, and improved activity of antioxidative enzymes in sciatic nerves [315]. Regarding cognitive functions, morin applied at doses of 50 and 100 mg/kg for 60 days reduced the escape latency time in the MWM task in rats treated with streptozotocin. This behavioral improvement was accompanied by reduced oxidative damage of proteins and membrane lipids in the brain, inhibition of apoptosis, increased BDNF levels, and regulation of the TrkB/Akt pathway [316].

**Figure 2:**
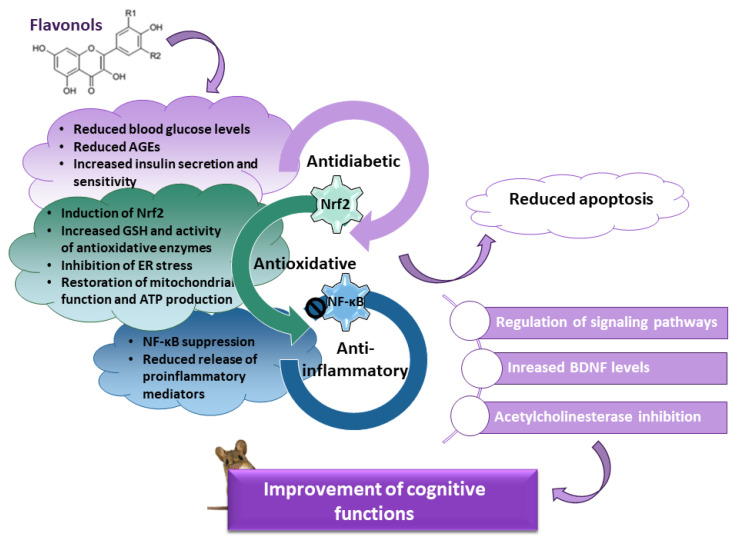
The main mechanisms contributing to the memory-enhancing effects of flavonols.

The main mechanisms contributing to the observed beneficial effects of flavonols on the improvement of cognitive abilities in preclinical models are summarized in Figure 2 and Table 1.

## 5. Conclusions

The pharmacological interventions in type 2 diabetes are mainly based on drugs that stimulate insulin secretion or sensitivity to insulin. The long-term intake of these drugs often results in side effects, indicating the necessity for alternative approaches. In that regard, herbal remedies emerged as an interesting therapeutic option, both in the prevention and treatment of diabetic complications.

Without going into the details of all the pathological mechanisms that are involved in the development of diabetes and its complications, oxidative stress and neuroinflammation are recognized as key factors contributing to cognitive decline in experimental models of diabetes. Accordingly, the improvement of antioxidative defense mechanisms and attenuation of the inflammatory response by targeting the Nrf2 and Nf-κB pathways hold great potential for slowing down the vicious cycle of molecular and cellular changes that result in hippocampal damage and cognitive decline. As oxidative stress has an important role in the activation of the NF-κB signaling cascade, the antioxidant strategy also alleviates the neuroinflammatory response. However, clinical trials with classic antioxidants like vitamins C and E were not very successful, suggesting that antioxidants showing multimodal mechanisms of action should be considered for pharmacological interventions.

In preclinical studies, there is a recognizable pattern of molecular and cellular mechanisms of action that are common to all flavonols with memory-enhancing properties. In general, they reduce levels of oxidative and inflammatory mediators, stimulate enzymatic and non-enzymatic systems of the endogenous antioxidative defense, increase levels of neurotrophic factors such as BDNF and NGF, restore cholinergic transmission, and regulate redox-sensitive and insulin-dependent signaling pathways. The combination of these synergistic effects results in an improvement in cognitive performance in behavioral tasks evaluating spatial and working memory. Although findings demonstrating the memory-enhancing properties of flavonols in experimental animals are tremendously encouraging, studies revealing the potential of flavonols in combating cognitive dysfunction in diabetic patients still need to be conducted. The effects of flavonols on other types of memories and other aspects of cognitive functioning should be investigated as well. A few studies have been conducted indicating that co-treatment with flavonoids from the two different classes could be a more efficient therapeutic strategy for obtaining maximal memory-improving effects, and further studies are warranted to answer this question. In addition to combining the effects of the two different flavonoids, the other approach for improving efficacy, especially at lower doses of flavonols, is based on the development of encapsulated forms that can more easily cross the blood-brain barrier. The improved delivery, together with molecular modeling and powerful bioinformatic tools used to advance the discovery of new drug candidates with flavonol structures, may help in obtaining better therapeutic efficacy and antioxidant and antiinflammatory activities of novel flavonols. On the other hand, in addition to psychometric tests, more comprehensive approaches based on neuroimaging techniques should be used to evaluate the potential of flavonols as a pharmacological intervention to restore cognitive abilities in diabetic patients.

Although several studies have provided encouraging results showing that dietary flavonol intake may be effective in preserving cognitive abilities in humans, the lack of clinical evidence on the nootropic effects of flavonols in diabetic patients should be an incentive for further clinical studies.

## Figures and Tables

**Table 1:** Effects of flavonols associated with the improvement of cognitive functions in diabetic animals.

Flavonol	Experimental Model	Route of Administration, Dose, and Treatment Duration	The Mechanisms Contributing to the Improvement of Cognitive Functions in the Neuronal Tissue	Reference(s)
Quercetin	STZ-induced diabetic rats	p.o., 5, 25 and 50 mg/kg/day, for 40 days	↓ MDA levels ↓ ADA activity ↓ AChE activity ↑ NTPDase activity	[239]
	female diabetic (*db*/*db*) mice	p.o., 70 mg/kg/day, for 12 weeks	↑ expression of synapse-related proteins (PSD93, PSD95) ↑ neurotrophic factors (BDNF, NGF) ↑ SIRT1 protein expression ↓ expression of ER stress markers (PERK, IRE-1α, ATF6, eIF2α, BIP, and PDI) ↓ oxidative stress levels	[240]
	STZ-induced diabetic rats	p.o., 30 and 60 mg/kg/day, for 6 weeks	restoration of the mitochondrial energy metabolism ↑ ATP production ↑ pAMPK, PGC-1α, SIRT1, NRF1, and TFAM expression ↑ AMPK/PGC-1α pathway	[236]
	High-fat diet- and STZ-induced diabetic rats	i.p., 50 mg/kg/day, for 14 days	↓ P2X_4_ receptor expression ↓ P2X_4_ and GFAP coexpression ↓ p38MAPK pathway ↓ p-p38MAPK	[243]
	STZ-induced diabetic rats	p.o., 25 mg/kg/day quercetin or QCSPIONs, for 35 days	normalized total antioxidant capacity ↓ miR-27a expression ↑ Nrf2 and expression of responsive antioxidant genes	[229,245]
	Diabetic (*db*/*db*) mice	p.o., 70 mg/kg/day, for 12 weeks	↑ expression of synapse-related proteins (PSD93, PSD95) ↑ neurotrophic factors (BDNF, NGF) ↑ SIRT1 expression ↓ expression of NLRP3 inflammation- related proteins ↓ NLRP3, adaptor protein ASC ↓ cleaved caspase-1, ↓ expression of pro-inflammatory cytokines IL-1β and IL-18 ↓ NLRP3 inflammasome activation ↓ expression of proapoptotic proteins	[246]
Rutin	STZ-induced diabetic rats	p.o., 100 mg/kg/day, for 5 weeks	↑ BDNF and NGF levels ↑ GSH levels ↓ lipid peroxidation antiapoptotic effect ↓ caspase-3 levels ↑ Bcl-2	[262]
Troxerutin	STZ-induced diabetic rats	p.o., 150 mg/kg/day, for 6 weeks	↓ lipid peroxidation ↓ oxidative stress ↑ SOD activity ↓ expression of NADPH oxidase subunits ↑ nuclear translocation of Nrf2 ↑ cytosolic fraction of HO-1 and NQO1	[263]
	STZ-induced diabetic rats	i.p., 60 mg/kg/day, for 6 weeks	↑ SOD activity ↑ GSH level ↑ GCLM and GCLC subunits expression ↓ MDA level	[264]
	STZ-induced diabetic rats	i.p., 60 mg/kg/day, for 12 weeks	↑ Nrf2 expression ↑ SOD activity ↓ lipid peroxidation	[265]
Myricetin	STZ-induced diabetic rats	i.p., 0.5, 1 or 2 mg/kg/day, for 2 weeks	↓ generation of AGEs and ROS ↑ Na+, K+-ATPase activity ↑ activity of antioxidative enzymes ↑ H_2_S, HO-1 and Nrf2 levels ↑ Nrf2 pathway	[278]
	STZ-induced diabetic rats	i.p., 5 or 10 mg/kg/day, for 21 day	↑ number of hippocampal CA3 pyramidal neurons	[279]
Dihydromyricetin	High-sugar, high-fat, and STZ-induced diabetic mice	p.o., 125 or 250 mg/kg/day, for 16 weeks	↓ oxidative stress ↓ MDA accumulation ↑ SOD, catalase and GPx ↑ BDNF	[284]
Morin	STZ-induced diabetic rats	50 mg/kg/day, for 60 days	↓ oxidative damage of proteins and membrane lipids ↓ apoptosis ↑ BDNF levels ↑ TrkB/Akt pathway	[306]

↑—increase; ↓—decrease; AChE—acetylcholinesterase; ADA—adenosine deaminase; AGEs—advanced glycation end-products; ATF6—activating transcription factor 6; BDNF—brain-derived neurotrophic factor; eIF2α—eukaryotic initiation factor 2*α*; ER—endoplasmic reticulum; HO-1—heme oxygenase-1; i.p.—intraperitoneal; IRE-1α— inositol-requiring transmembrane kinase/endoribonuclease 1α; GFAP—glial fibrillary acidic protein; GCLC—glutamate-cysteine ligase catalytic; GCLM—glutamate-cysteine ligase modifier; GPx—glutathione peroxidase; GSH—glutathione; MDA—malondialdehyde; NADPH—nicotinamide adenine dinucleotide phosphate; NGF—nerve growth factor; pAMPK—phosphorylated AMP-activated protein kinase; PDI—protein disulfide isomerase; PERK—protein kinase R-like endoplasmic reticulum kinase; PGC-1α—peroxisome proliferator-activated receptor-gamma coactivator-1alpha; NLRP3—NOD-, LRR- and pyrin domain-containing 3; NRF1—nuclear respiratory factor 1; Nrf2—nuclear factor erythroid 2–related factor 2; NQO1—NAD(P)H:quinone oxidoreductase; p.o.—per os; PSD93—postsynapticdensity 93; PSD95—postsynapticdensity 95; p38MAPK—p38 mitogen-activated protein kinase; p-p38MAPK—phosphorylated p38 mitogen-activated protein kinase; QCSPIONs—quercetin conjugated with superparamagnetic iron oxide nanoparticles; ROS—reactive oxygen species; SOD—superoxide dismutase; STZ—streptozotocin; TFAM—mitochondrial transcriptional factor A.

## Data Availability

Not applicable.
